# The secret lives of *Drosophila* flies

**DOI:** 10.7554/eLife.06793

**Published:** 2015-06-04

**Authors:** Therese Ann Markow

**Affiliations:** Division of Biological Sciences, University of California at San Diego, La Jolla, United States and Laboratorio Nacional para Genomica de la Biodiversidad, Irapuato, Mexico

**Keywords:** the natural history of model organisms, species, natural history, ecology, *D. melanogaster*

## Abstract

Flies of the genus *Drosophila*, and particularly those of the species *Drosophila melanogaster*, are best known as laboratory organisms. As with all model organisms, they were domesticated for empirical studies, but they also continue to exist as wild populations.

Decades of research on these flies in the laboratory have produced astounding and important insights into basic biological processes, but we have only scratched the surface of what they have to offer as research organisms. An outstanding challenge now is to build on this knowledge and explore how natural history has shaped *D*. *melanogaster* in order to advance our understanding of biology more generally.

**DOI:** http://dx.doi.org/10.7554/eLife.06793.001

## Introduction

From its first use in the laboratory in the early 1900s until the present day, *Drosophila melanogaster* has been central to major breakthroughs in genetics. The use of this fruit fly as a model organism began with the pioneering work of Thomas Hunt Morgan, who was awarded the 1933 Nobel Prize in Physiology or Medicine for ‘his discoveries concerning the role played by the chromosome in hereditary’*.* Morgan's former student, Herman J Muller, subsequently received the prize in 1946 ‘for the discovery of the production of mutations by means of X-ray irradiation’. In 1995, the *Drosophila* researchers, Edward B Lewis, Christiane Nüsslein-Volhard and Eric F Wieschaus shared the prize ‘for their discoveries concerning the genetic control of early embryonic development’. Most recently, Jules Hoffman shared the 2011 prize for ‘discoveries concerning the activation of innate immunity’ in *Drosophila*.

How did one species of *Drosophila*, *D. melanogaster*, come to be a model system? Harvard entomologist Charles Woodworth was the first to rear *D. melanogaster*, just after the turn of the 20th century*.* It is not clear why or how he came to breed them, but their short generation time and ease of rearing were probably very appealing attributes. Woodworth then recommended them to his colleague William Castle, who initially worked on mammals but utilized the flies to study inbreeding. During this same period, another entomologist, Frank Lutz at the American Museum of Natural History, also began studying this fly's basic biology, publishing more than a dozen papers about them (Davenport, 1941; Carlson, 2013). It was from Lutz that Thomas Hunt Morgan introduced them into his laboratory at Columbia University. At the time Morgan began his work, the basic principles of heredity were still under debate. Morgan's discoveries and the fact that he attracted a highly talented group of graduate students no doubt fuelled the use of *D. melanogaster* as a model system.

But what do we know about the biology of this fly in nature? Here, I review what we know of its origins, its biology in the wild and how this differs from what we see in the laboratory, its natural history, and why its natural history matters for laboratory studies, as well as its advantages as a model organism. I also discuss why, even after so many years of intensive investigation, *D. melanogaster* and its relatives are in an important position to help us address central questions about biology.

## Where did *D. melanogaster* come from and how do these flies live?

*D. melanogaster,* described by Meigen in 1830, appears to have originated in sub-Saharan Africa (Lachaise et al., 1988). The first out-of-Africa habitat expansion of *D. melanogaster* is thought to have occurred between 10,000 and 15,000 years ago, when it moved to Europe and Asia (David and Capy, 1988). North America and Australia were colonized more recently (David and Capy, 1988). Subsequent colonization events, especially as human travel has accelerated, have continued to move populations around the globe. Its current distribution is worldwide, being found on every continent and most islands (Markow and O'Grady, 2005b).

A human commensal associated primarily with rotting fruits, *D. melanogaster* is also associated with a wide array of decaying vegetables and other plant matter. The fact that this fly is an ecological generalist no doubt contributed to the facility with which it was initially propagated in the laboratory, rapidly becoming a popular model system. *Drosophila* are found worldwide, and their extensive distribution has allowed studies of adaptations to different latitudes.

*D. melanogaster* do not live alone. Their decaying host resources are also home to many microbes, as well as to other arthropods, including other *Drosophila* species, all of which they interact with (see Video 1, 2). Some microbes in the decaying material themselves provide food for *D. melanogaster*, being selectively consumed by larvae or adults. Other microbes are essential for decomposing fruit and other plant matter into substances, such as volatiles, that attract other adult flies to the food source, or for decomposing organic matter into new material, which in turn is consumed by the flies. Along with *Drosophila simulans, Drosophila hydei, Drosophila immigrans*, and *Drosophila busckii*, *D. melanogaster* forms part of what is known as the ‘cosmopolitan guild’ of *Drosophila* (Atkinson and Shorrocks, 1977)*.* While found in association with these other species, *D. melanogaster* colonizes the rotting fruits at a particular time during the decay trajectory. First to arrive is *D. simulans,* followed by *D. melanogaster,* and then the other species (Nunney, 1990, 1996): this is consistent with *D. melanogaster* having a higher ethanol tolerance than its relative *D. simulans* (McKenzie and Parsons 1972, 1974), which arrives earlier, when fewer volatiles have been produced by fermentation. Other arthropods, especially beetles, are also common in the substrate and are predators of the developing flies.

**Video 1.** Three members of the cosmopolitan guild of *Drosophila* feeding. *D. hydei* (the larger, dark flies) and *D. melanogaster* and *D. simulans* (the smaller, lighter flies) quietly feeding on the juice of a rotting tomato and on the microbes present on it. *D.* *melanogaster* and *D. simulans* are sibling species and are morphologically indistinguishable in the video. Video credit: Therese Ann Markow. **DOI:** http://dx.doi.org/10.7554/eLife.06793.002

**Video 2.** Three members of the cosmopolitan guild of *Drosophila* interacting at a food source. *Drosophila*: *D. hydei* (the larger, dark flies) and *D. melanogaster* and *D. simulans* (the smaller, lighter flies) interacting at a food source. Although there is little sexual dimorphism between males and females of the *D. hydei* species, males can be distinguished in the video because they approach other flies to court. In the *D. melanogaster* and *D. simulans* species*,* males are smaller than the females and have darker abdomens. These males can also be seen approaching other flies and attempting to court. Attempted courtships are brief and often end when females extrude their ovipositors. Notice that males will approach flies of different sexes and species, and that flies of *D. hydei* are much less active than those of *D. simulans* and *D. melanogaster.* *D.* *melanogaster* and *D. simulans* are sibling species and are morphologically indistinguishable in this video. Video credit: Therese Ann Markow. **DOI:** http://dx.doi.org/10.7554/eLife.06793.003

**Figure 1.**
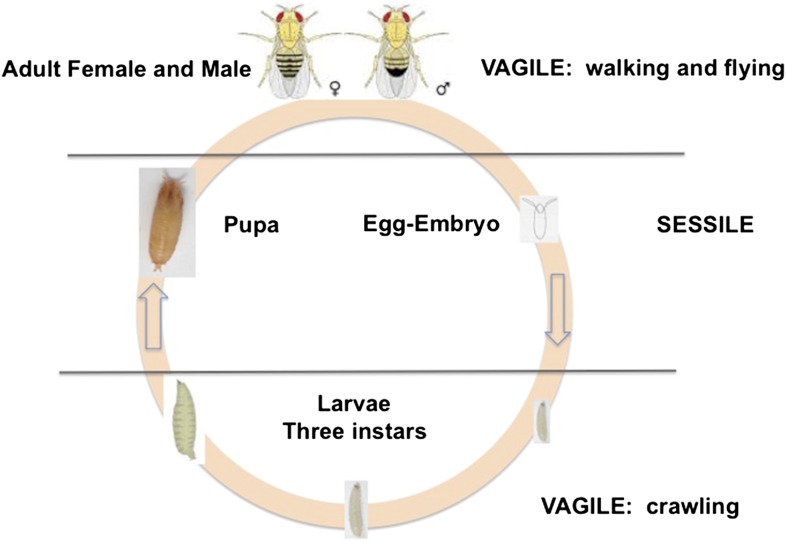
The life cycle of *Drosophila melanogaster*. Egg and pupa stages are sessile, larvae move within the substrate, and adults are highly vagile as their ability to fly enables their dispersal. Different species of Drosophila vary in their larval development times, as well as in the ages at which females and males attain reproductive maturity. Image credit: Therese Ann Markow. **DOI:** http://dx.doi.org/10.7554/eLife.06793.004

*D. melanogaster* is holometabolous, meaning it undergoes a metamorphosis from its larval to adult form (Figure 1). Females lay their eggs in necrotic material, and the larvae develop and pupate there. Two life stages are completely immobile: the egg and the pupa. Larvae can move within the resource patch, while adults can fly between patches. Given the sessile status of eggs and larvae, we expect these stages to exhibit adaptations against predation, parasitism and environmental stressors, such as temperature extremes, ultraviolet light and desiccation. Natural selection on behaviors such as oviposition and pupation site selection is therefore expected to be strong.

## What is different in the lab and field?

In the laboratory, life is simple. Much is constant. Flies are grown in one or more standardized culture media, usually treated with mould inhibitors, such as propionic acid or methylparaben, and antibiotics. While these culture conditions keep flies ‘healthy’ by laboratory standards, they do not represent the conditions that *D. melanogaster* experience in nature. The fungal, bacterial and viral pathogens (Magwire et al., 2012; Keebaugh and Schlenke, 2014) flies encounter in nature are absent in the laboratory. In the wild, larvae and flies are also exposed to predators, such as ants, beetles, pseudoscorpions and lizards, as well as to parasites, such as wasps and phoretic mites. Encountering food of different types and ages in nature also differs from the benign consistency of the laboratory environment. In the laboratory, flies tend to be reared at a constant temperature and humidity level, while these abiotic variables fluctuate in nature. Laboratory adults also don't need to disperse to find a resource for the next generation.

What is different then, about the flies themselves, when found in nature? Few studies of *D. melanogaster* have been done in the wild, but those that have reveal a different picture of wild flies. For one thing, they tend to be larger than laboratory reared flies, possibly owing to some, as yet unknown, micronutrients and/or to the fact that in nature, temperatures fluctuate and growth is slower than in culture (Chown and Gaston, 2010). The microbes that associate with *D. melanogaster* in the laboratory are also far less diverse than those that associate with these flies in the wild (Chandler et al., 2011).

Reproductive behavior and biology, while extensively studied in the laboratory, is less well-understood in the wild. From the few studies conducted in nature, a different picture emerges. For example, in nature, virgins are not separated upon eclosion and stored until used in experimental pairings. Instead, they tend to be mated early and often (Markow et al., 2012). In fact, many *D. melanogaster* females in the wild appear to have been force-mated by males waiting for them to emerge from their pupa cases (Markow, 2000; Seeley and Dukas, 2011). In addition, while laboratory mated females tend to die earlier than do virgins, the well known ‘cost of mating’ (Fowler and Partridge, 1989), in nature the opposite seems to be true (Markow, 2011). Courtship itself is also different in nature compared to that observed in the laboratory. Laboratory experiments almost universally reveal an advantage to large males when placed with smaller males in ‘choice’ experiments (Alcock, 2013). In nature, however, sexual interactions do not take place in small chambers. Males appear to sort themselves out by size at the mating site, with smaller males often being found in parts of the fruit where there are fewer females and thus fewer matings (Markow, 1988). The mating advantage to larger males is not as apparent in wild populations (Partridge et al., 1987; Markow, 1988). Furthermore, when courted by an undesirable male in nature, where there is ample space to escape, female *D. melanogaster* rarely decamp, instead, extruding their ovipositor to discourage the suitor (Gromko and Markow, 1993; Video 2).

## Untapped potential of *Drosophila*

Our extensive foundational knowledge of the biology of *D. melanogaster* places these flies in a very strong position to contribute to our understanding of outstanding issues and questions in biology, supported by the availability of a sequenced genome (Adams et al., 2000) and an array of genomic resources. While a number of future discoveries will concern basic processes in gene action and development, the natural history of *D. melanogaster* can also inform and guide discoveries relevant to contemporary and pressing problems in human health and environmental change.

### Reproduction and biocontrol

The reproductive systems of *Drosophila* species are among the most variable of any organism (Markow, 1996, 2002; Markow and O'Grady, 2005a). Some of this variability is behavioural. For example, in some species, such as *D. hydei* and *Drosophila nigrospiracula,* females will mate multiple times in a single morning, while in others, such as *Drosophila subobscura,* females will mate once in their lifetime. Some species, such as *Drosophila pachea,* require weeks for an adult fly to become sexually mature, while in others, such as *Drosophila mettleri,* either sex can be ready to mate within hours of emerging from the pupa case. The genes that control these behavioural differences can hold clues to controlling the reproduction of economically and medically important insects, such as testse flies and mosquitoes (see Box 1). Morphological variation can also influence the reproductive success of ‘problem’ species. *Drosophila suzukii*, for example, is an exceptional species that has recently invaded America and Europe from Asia (Rota-Stabelli et al., 2013) and attacks agricultural produce (in particular, by laying its eggs into soft fruits). A sequenced genome (Chiu et al., 2013) and comparative morphological studies of its females' sharp ovipositor (Atallah et al., 2014) provide insights into the basis for its rapid invasion.

10.7554/eLife.06793.005

Box 1. **Outstanding questions about the natural history of *Drosophila***

- Why can some *Drosophila* species feed and breed in certain resources while other species cannot?
- Why can some *Drosophila* species tolerate extreme environmental conditions while others cannot?
- What accounts for the particular microbial communities found inside the guts of *D. melanogaster* and of other species?
- What accounts for the astounding variability in the reproductive biology of *Drosophila* species?

**DOI:** http://dx.doi.org/10.7554/eLife.06793.005

An additional aspect of biocontrol is to understand the neurobiological mechanisms by which insects identify their hosts. Here again, discoveries made in *D. melanogaster* can be applied to economically important species. These discoveries include the first functional mapping of olfactory responses (Hallem and Carlson, 2004), and the use of multiple species' genomes to reveal the ecological and behavioural significance of the evolution of various olfactory receptors (McBride, 2007; Guo and Kim, 2007; Goldman-Huertas et al., 2015). As such, the *D. melanogaster* toolbox can now be used to disrupt host-seeking behaviors in insects of medical and economic importance (Carey and Carlson, 2011).

### Human health

*D. melanogaster* has played an increasingly important role in the creation of animal models of human disease. Approximately 65% of human disease genes are estimated to have counterparts in *D. melanogaster* (Chien et al., 2002), most of which are available in the Homophila database (http://superfly.ucsd.edu/homophila/). The number of investigators using *D. melanogaster* as a model for studying human disease is steadily rising (Pfleger and Reiter, 2008), especially for more complex disorders, such as heart disease (Piazza and Wessells, 2011), mental and neurological illness (Pandey and Nichols, 2011), and obesity (Trinh and Boulianne, 2013).

Complex health problems tend to be rooted in the interaction between multi-factorial genotypes and the environment. What role can natural history play in our ability to understand these interactions with a view towards disease mitigation and treatment? In the past few decades, the importance of the gut microbiome for models of human health has grown. *The D. melanogaster* microbiome, under laboratory conditions, turns out to be quite simple, with an average of ten culturable bacterial species (Lee and Hase, 2014), and has provided insights into the relationship between gut microbiota and processes such as intestinal function (Lee and Lee, 2014) and insulin signaling (Shin et al. 2011). Of considerable interest is that the microbiome of wild *D. melanogaster* is much more complex (Cox and Gilmore, 2007) than that found in laboratory reared flies, comparable to the differences observed between non-westernized human populations and urban populations that consume highly processed diets (De Filippo et al., 2010) (see Box 1). This similarity between flies and humans reveals the importance of host-microbiota homeostasis for human health (David et al., 2014; Kostic et al., 2013). For example, Shin et al. (2011) demonstrated how the *Drosophila* gut microbiome regulates the metabolic homestasis of the fly.

### Global environmental change: detoxification and stress resistance

Environmental change is actually a complex of changes, both abiotic and biotic. Abiotic challenges include changing temperature and humidity, and biotic challenges, often fomented by abiotic shifts, include changes in available habitat, presence of pathogens, parasites, competitors and invaders. Understanding adaptation to global environmental change thus also is a complex problem, and one that requires us to monitor natural populations, as well as to conduct laboratory studies to discover the bases of adaptations or the lack thereof. Natural populations and laboratory strains of *D. melanogaster* have been successfully exploited in examining responses to changing environments (Rodríguez-Trelles and Rodríguez, 2007; Hoffmann, 2010). The susceptibility of *D. melanogaster* to global environmental change is well documented in the clinal or seasonal changes in the frequencies of alleles at particular loci (Umina et al., 2005) and in changes in chromosomal inversion frequencies (Anderson et al., 2003). Several thousand *Drosophila* species, some with highly specialized ecologies, are limited in their distributions to very cold or very hot climates. For example, *D. pachea* is endemic to the Sonoran Desert of North America, where it depends on the sterols in the cactus *Lophocereus schottii,* which has alkaloids that other *Drosophila* species cannot tolerate. Because of its obligate association with its cactus host, it is exposed to temperatures that often approach 50°C. Such species provide unprecedented opportunities to understand the genetic bases of adaptations to extreme situations (see Box 1) and to recruit these species to address problems of species loss in the face of global warming and other anthropogenic changes.

Another product of anthropogenic change is the evolution of pesticide resistance in a wide range of insects of economic and medical importance. Natural and laboratory populations of *D. melanogaster* have played key roles in our understanding of the roles of the cytochrome P450-encoding genes and the glutathione *S*-transferases in resistance to the insecticide, dichlorodiphenyltrichloroethane (better known as DDT) (Ffrench-Constant, 2013). Various other *Drosophila* species have specialized on resources (such as cacti or *Morinda* fruit) that contain a range of allelochemicals, or secondary metabolites, many of which are toxic to other organisms and thus serve as defense against herbivory. The genetic bases of these specializations, as they relate to phenomena such as the evolution of pesticide resistance (McDonnell et al., 2012; Miyo, 2012) and detoxification (Gloss et al., 2014; Mitchell et al., 2014), are already being investigated through comparative genomics.

## Combining genomics and natural history

**Figure 2.**
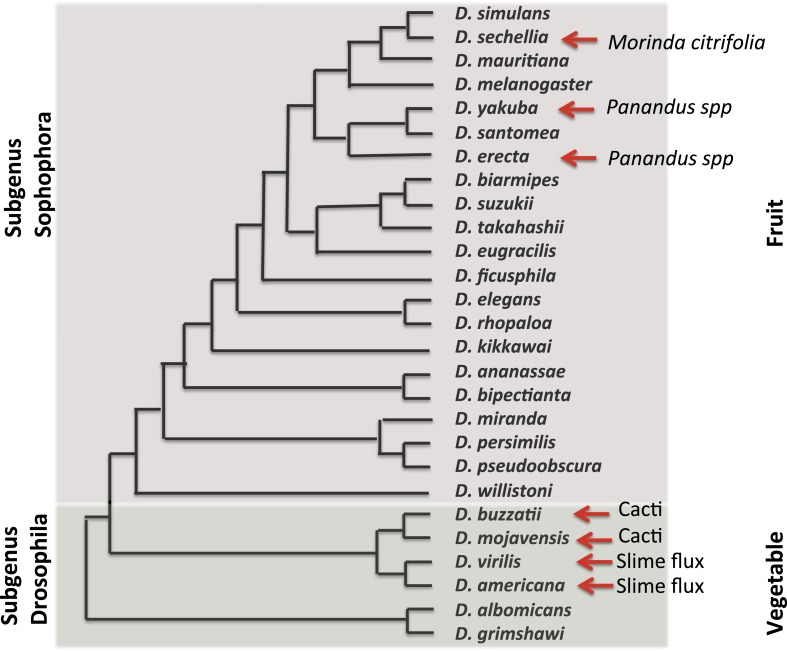
Evolutionary and ecological relationships of Drosophila species. Phylogenetic relationships (based on Markow and O'Grady, 2005b) are shown for species with available assembled whole-genome sequences. Within the subgenus Sophophora, *D. sechellia* has specialized to consume and breed on Morinda fruit and *D. erecta* has similarly specialized on fruits of various Panandus species, as has *D. yakuba* although to a lesser degree. Within the subgenus Drosophila, *D. buzzatii* and *D. mojavensis* breed in cacti, while *D. virilis* and *D. americana* breed in the slime fluxes of deciduous trees. Even among specialists, adult flies may feed more broadly while larvae are more specialized. Arrows indicate substrate specialization by these species. Image credit: Therese Ann Markow. **DOI:** http://dx.doi.org/10.7554/eLife.06793.006

In 2003, the fly community submitted a white paper for the whole-genome sequencing of additional *Drosophila* species. The resultant 2007 publication by the Drosophila 12 Genomes Consortium et al., 2007, of 12 genomes and their analysis, has rapidly revolutionized and expanded the utility of the *Drosophila* system for studies ranging from computational biology and embryology, to evolution and human disease. In the short time since these genomes were made available, insights have been gained into the emergence and loss of new pathways, the gain and loss of pathway complexity (Salazar-Jaramillo et al., 2014), and the changes in the regulatory network of complex genomes (Coolon et al., 2014; McManus et al., 2014). At this point in time, over 30 *Drosophila* genomes have been sequenced, further expanding the importance of and opportunities provided by these flies. These species, their evolutionary relationships and ecological features, are presented in Figure 2.

The natural histories of these species are diverse. Some are highly specialized and live under extreme climatic conditions. Others are adapted to diets high or low in protein or carbohydrates. Their microbiomes differ (Chandler et al., 2011) as does their genomic machinery for dealing with various environmental challenges (Low et al., 2007; McDonnell et al., 2012).

## Conclusions

The expanding number of sequenced *Drosophila* species' genomes offers a tremendous opportunity to learn from the ways in which different species have solved the challenges of living in different niches. But laboratory studies alone, in the absence of an understanding of the natural history, the challenges and lifestyles of these flies, will never allow us to fully exploit what they have to offer. By characterizing the natural history of not just *D. melanogaster* but also of those other Drosophilids with contrasting ecologies, we will be able to detect and exploit such phenomena as novel resistance mechanisms and novel dietary adaptations and reproductive strategies.

This knowledge can then be employed to advance our understanding of basic biological principles, thus building a more robust toolbox to apply to human problems. For example, the efficacy of anticancer therapeutic agents depends not only on their effects on the tumor but also on the ability of the host to tolerate the toxic effects of the drug. The many ways in which fly species have dealt with detoxification and tolerance could inform and refine drug discovery. It's not difficult, as one might imagine, to study a large number of different *Drosophila* species in the wild. But it's time to do more of it.
